# The critical roles of mitophagy in cerebral ischemia

**DOI:** 10.1007/s13238-016-0307-0

**Published:** 2016-08-24

**Authors:** Yan-Cheng Tang, Hong-Xia Tian, Tao Yi, Hu-Biao Chen

**Affiliations:** 1 School of Chinese Medicine, Hong Kong Baptist University, Kowloon Tong, Hong Kong China; 2 Medical Research Department, Guangdong General Hospital and Guangdong Academy of Medical Sciences, Guangzhou, 510080 China; 3 HKBU Institute of Research and Continuing Education, Shenzhen, 518057 China

**Keywords:** autophagy, mitophagy, mitochondria, cerebral ischemia

## Abstract

Mitochondria play a key role in various cell processes including ATP production, Ca^2^^+^ homeostasis, reactive oxygen species (ROS) generation, and apoptosis. The selective removal of impaired mitochondria by autophagosome is known as mitophagy. Cerebral ischemia is a common form of stroke caused by insufficient blood supply to the brain. Emerging evidence suggests that mitophagy plays important roles in the pathophysiological process of cerebral ischemia. This review focuses on the relationship between ischemic brain injury and mitophagy. Based on the latest research, it describes how the signaling pathways of mitophagy appear to be involved in cerebral ischemia.

## **INTRODUCTION**

Stroke is one of the leading causes of death worldwide and is a major cause of adult disability. Cerebral ischemia is a common form of stroke caused by insufficient blood supply to the brain. According to which areas of the brain are affected, there are two types: global ischemia, and focal ischemia. Repression of blood supply to the entire brain causes global ischemia while occlusion of certain cerebral blood vessels causes focal ischemia. The main symptoms of cerebral ischemia are sudden loss of consciousness, blindness and coordination defects, including speaking problems. Biochemically, there are profound reductions in ATP levels for very short periods during ischemia, possibly for as little as 1–2 min. Nevertheless, there is massive cell death in vulnerable regions. The critical factor is blood flow. If blood flow is restored to the affected tissue soon enough, cerebral ischemic damage may be reversible; however, if blood flow is not restored, the tissue dies resulting in irreversible damage. Currently, there are two therapeutic strategies. The most common, used in early cerebral ischemia, is to restore blood flow to the affected area of the brain as quickly as possible by administering alteplase, aspirin and anticoagulants drugs. Nevertheless, all of these drugs have some side effects in that they can cause a second wave of damage, a consequence termed reperfusion injury. Another therapeutic strategy is neuroprotection, which is intended to save the penumbral tissues and to extend the time window for revascularization techniques. However, at the present time, there are no known neuroprotective agents, and mechanisms of neuroprotection strategy are still unclear. Thus understanding why this cell death arises from such short insults is a major goal of research in the field. Researchers have identified at least three recognizable pathways of ischemic cell death: necrotic, apoptotic, and autophagocytotic cell death (Lipton, 1999). Different from apoptosis and necrosis, which certainly lead to ischemic brain injury, autophagy possibly serves as a potential therapeutic target against ischemic brain injury (Carloni et al., 2010).

Autophagy is an evolutionarily conserved process by which lysosomes degrade unnecessary or dysfunctional proteins and cell organelles. The process of autophagy was first observed by Ashford and Porter in 1962, when they found that cells could eat themselves (Ashford and Porter, 1962). The term “autophagy” was first coined by De Duve, who also established that lysosomes were responsible for glucagon-induced autophagy (Deter et al., 1967; Deter and De Duve, 1967). During autophagy, unnecessary or dysfunctional cellular components are engulfed by a double-membraned vesicle known as an autophagosome. Then the autophagosome is fused by lysosome, which leads to the degradation and the recycling of the cellular components and proteins. In mammalian cells, there are three types of autophagy: macroautophagy, microautophagy, and chaperone-mediated autophagy (CMA). Macroautophagy is the most common and well-studied form, and, typically, when general term “autophagy” is used it refers to macroautophagy. Hence, in this rest of this review, the term “autophagy” should be taken as referring to macroautophagy. While autophagy has been known for more than fifty years, its mechanisms have only come to be understood in the last decade. During this period of explosive growth in understanding, in addition to classical autophagy, at least five types of selective autophagy have been identified, namely mitophagy, xenophagy, pexophagy, ribophagy, and reticulophagy. Both classical autophagy and selective autophagy are very important for cell and tissue homeostasis, and they are involved in the natural process of aging as well as many human diseases, including muscular dystrophy, cancer, and innate immunity and neurodegenerative disorders (Mizushima and Komatsu, 2011).

The roles of autophagy in the cerebral ischemia process have been widely studied. Recent reports have showed that autophagy can be induced in both *in vitro* (Meloni et al., 2011) and *in vivo* (Tian et al., 2010) cerebral ischemia models. In cerebral ischemia injury, autophagy is a double-edged sword; it can be protective (Carloni et al., 2010) or destructive (Koike et al., 2008). If its protective functions can be controlled, autophagy could be a valuable therapeutic target. However, the roles of selective autophagy in this process are unclear and reviews are limited. In the past five years, more and more groups have focused on the relationship between cerebral ischemia and selective autophagy. Mitophagy is one well-studied type of selective autophagy which is extremely important for maintaining mitochondria homeostasis by removing damaged mitochondria. Mitochondria are called the “the powerhouses of the cell” and are involved in cell signaling, cellular differentiation, and cell death, as well as maintaining control of the cell cycle and cell growth. Mitochondrial dysfunctions are related to many diseases, including diabetes, heart failure, innate immunity responses and neurological defects (Mizushima and Komatsu, 2011). In this review, we summarize the current knowledge on the regulation of mitophagy and its specific roles in cerebral ischemia and focus on the molecular mechanisms and pathophysiological roles that regulate mitophagy in ischemic brain injury.

## MITOPHAGY SIGNALING PATHWAYS AND RESEARCH METHODS

**Figure 1:**
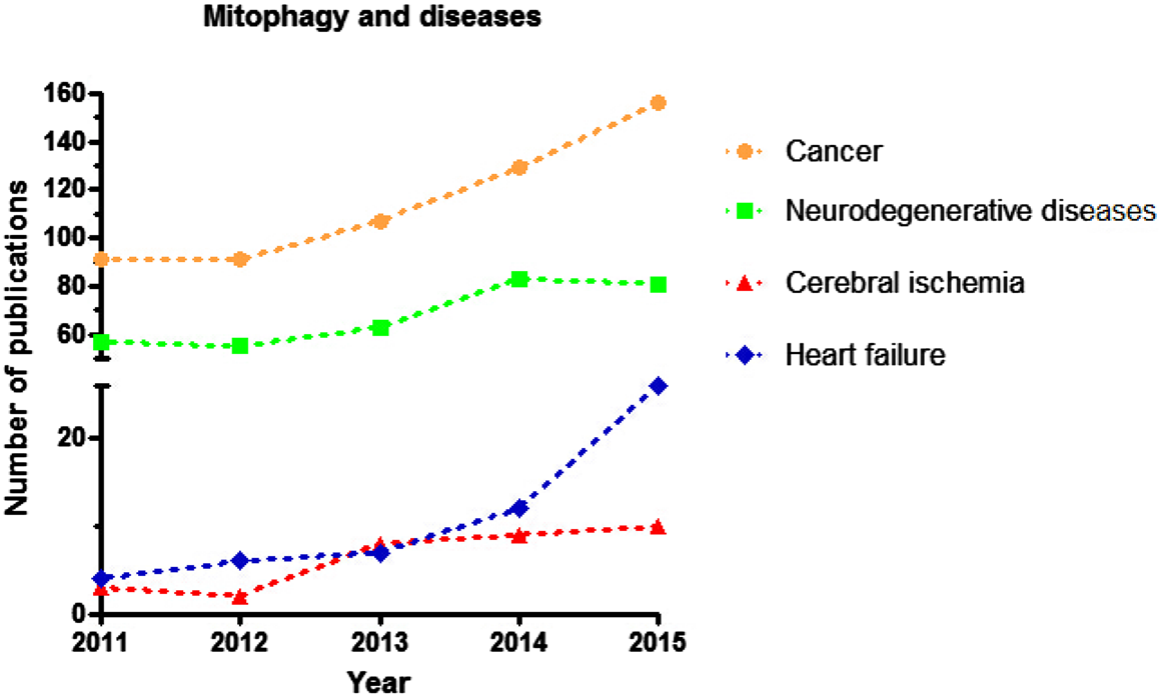
**Trends in research on mitophagy in human disease**. Summary of the study trends of mitophagy and its related human disease from 2011 to 2015

The name “mitophagy” was first coined by J.J. Lemasters in 2005. Lemasters and colleagues described how they found depolarized mitochondria engulfed by vesicles coated with the autophagosome marker MAP1 light chain 3 (LC3) when they treated rat hepatocytes with serum starvation (Park et al., 2006). While much is still unclear, in the past five years considerable progress has been made in understanding the molecular mechanisms of mitophagy and in determining its pathophysiological roles (Fig. 1). Serious study of the biochemical mechanism of mitophagy was first undertaken with yeast. Kissova et al. reported that Uth1p, which was mainly localized in the mitochondrial outer membrane (OMM) and contained a SUN domain, was required for mitophagy. During nutrient starvation, Uth1p was required for eliminating excess mitochondria (Kissova et al., 2004). Tal et al. reported that Aup1p was also required for mitophagy. Aup1 was a member of the phosphatase2c superfamily and localized to the mitochondrial intermembrane space; it facilitated mitophagy in stationary phase cells (Tal et al., 2007).

**Figure 2:**
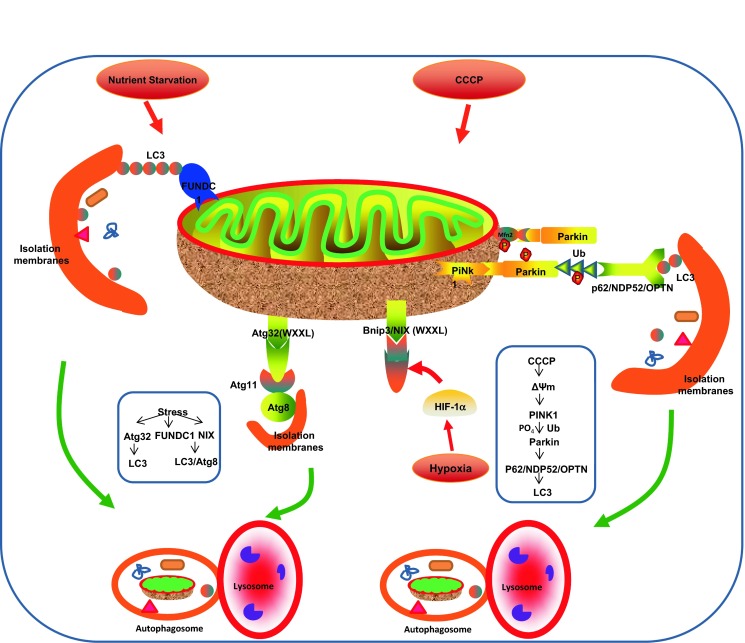
**Mechanisms of mitophagy**. (a) In yeast, the outer mitochondrial membrane protein Autophagy-related 32 (Atg32) binds the isolation membrane protein Atg8 through its WXXL-like motif. This process requires adaptor protein Atg11, which can form a complex with Atg32 and Atg8 and physically link mitochondria with isolated membranes. Finally, the mitochondria are sealed by the isolation membranes and fuse with lysosome to be degraded. Uth1p and Aup1p are both mitochondria membrane proteins and can facilitate mitophagy under certain special conditions, such as starvation. (b) In mammalian cells, (1) Parkin-dependent pathway. After treatment with CCCP or other mitochondria inhibitors, the mitochondria are damaged and lose membrane potential, which can lead to impaired PINK1 cleavage and stabilization. At that point, PINK1 phosphorylates Parkin and ubiquitin at Ser65 to recruit Parkin to damaged mitochondria from the cytosol. Parkin ubiquitylates the mitochondrial substrates and generates more ubiquitin substrate for PINK1 to phosphorylate; then, the ubiquitin-binding adaptor p62/NDP52/OPTN aggregates ubiquitylated proteins and recruits ubiquitylated cargo into autophagosome by binding to LC3. Finally, the mitochondria are sealed by the isolation membranes and fuse with lysosomes to be degraded. (2) Parkin-independent pathway. The most important Parkin-independent pathway is the NIX/Bnip3 and FUNDC1 pathway. Under hypoxic conditions or starvation, the protein level of NIX or Bnip3 increase. NIX and Bnip3 localize on the outer membrane of mitochondria and contain a WXXL-like motif facing the cytosol which can directly bind to the mammalian Atg8 orthologue and LC3, thereby facilitate mitophagy. FUNDC1 is a mitochondria outer membrane protein containing a classical LC3-interacting region. Activated FUNDC1 directly binds with LC3 or ATG8 to induce subsequent mitophagy

Mammalian homologues of Uth1p and Aup1p have not been found. In addition, mitophagy in mammalian presents some unique features. In general, the mechanisms of mitophagy in mammalian cells can be Parkin-dependent and Parkin-independent pathways. The possible mitophagy pathways are summarized in Fig. 2 and discussed in the following sections.

### PINK1-Parkin signaling pathway in mitophagy

PINK1, a 64-KD protein, is encoded by PTEN-induced kinase1, which contains a mitochondrial targeting sequence (MTS). Parkin is an E3 ubiquitin ligase. PINK1 and Parkin have been found to function in one pathway to suppress mitochondrial damage in flies. PINK1 may be an upstream regulator of Parkin (Clark et al., 2006; Park et al., 2006). *PINK1* and *Parkin* are often found mutated in Parkinson patients and they are suspected pathological causes of early onset of Parkinson’s disease, a process involving their regulatory role in mitophagy (Deas et al., 2011).

Parkin was firstly demonstrated to be involved in mitophagy in 2008. Narendra et al. found that Parkin normally resided in the cytosol but it could be translocated to depolarized mitochondria after one hour treatment of a mitochondria uncoupler, carbonylcyanide *m*-chlorophenylhydrazone (CCCP). Mitochondria-localized Parkin can recruit the autophagy marker LC3 to mitochondria, which promotes the degradation of mitochondrial by mitophagy (Narendra et al., 2008).

The translocation of Parkin is required for PINK1 activity. PINK1 shuttles between cytosol and mitochondria. In healthy cells, PINK1 is constitutively imported into mitochondria and cleaved by mitochondrial inner membrane rhomboid protease presenilin-associated rhomboid-like protein (PARL). But when the mitochondria are depolarized by CCCP, PINK1 can be stabilized on the outer membrane of mitochondria and recruits Parkin. In mouse cardiomyocytes, the recruitment of Parkin by PINK1 was mitofusin 2 (Mfn2, mitochondrial outer membrane guanosine triphosphatase) but not mitofusin 1 (Mfn1) dependent (Chen and Dorn, 2013). PINK1 stabilized in depolarized mitochondria phosphorylated Mfn2 to enhance the interaction between Parkin and Mfn2; Mfn2 served as a Parkin receptor on damaged mitochondria to promote the translocation of Parkin from cytosol to mitochondria to induce mitophagy (Chen and Dorn, 2013). However, Parkin could still translocate to mitochondria in cultured embryonic fibroblasts lacking Mfn2, which indicated other Parkin recruitment mechanisms exist (Chan et al., 2011; Wauer et al., 2015).

Indeed, PINK1 and Parkin could physically interact with each other, and Parkin could be phosphorylated on Thr175, Thr217 and Ser65 by PINK1; more recent efforts have revealed that this phosphorylation is required for Parkin’s translocation (Kim et al., 2008). Parkin is a RING in between RING (RBR) domain family of E3 ubiquitin ligases, containing an N-terminal ubiquitin-like (UBL) domain, RING1 domain, three zinc-coordinating domains and RING2 domain (Wauer et al., 2015). In cytosol, Parkin exists in an auto-inhibited conformation (Chaugule et al., 2011; Riley et al., 2013; Wauer and Komander, 2013). In this status, Parkin’s UBL domain as well as the repressor element block the E2-binding site in RING1 domain, and the catalytic C431 in RING2 is blocked by the unique Parkin domain (UPD, also known as RING0) (Chaugule et al., 2011; Riley et al., 2013; Wauer and Komander, 2013). After mitochondria depolarization, stabilized PINK1 phosphorylates Parkin at Ser65 in the UBL domain to activate Parkin by driving conformational changes (Shiba-Fukushima et al., 2012). Another mechanism that PINK1 activating Parkin is that PINK1 phosphorylates ubiquitin at Ser65 (pS65-Ub), and pS65-Ub binds and activates Parkin due to Parkin’s high affinity for pS65-Ub (Kane et al., 2014; Kazlauskaite et al., 2014; Wauer et al., 2015). When binding with Parkin, pS65-Ub can release the inhibitory UBL domain of Parkin from the RBR core and stretches the of the helix in the REP, leading to conformational changes to make an ‘open’ active Parkin (Wauer et al., 2015); releasement of the UBL domain also promotes the phosphorylation of Parkin at S65 by PINK1, which further activates Parkin (Kumar et al., 2015; Sauve et al., 2015; Wauer et al., 2015).

Parkin’s promotion of mitophagy relies on its E3 ubiquitin ligase activity. After treatment with CCCP, Parkin ubiquitylates the mitochondria proteins; then, the ubiquitin-binding adaptor p62 aggregates ubiquitylated proteins and recruits ubiquitylated cargo into autophagosome by binding to LC3; finally, the mitochondria are degraded by lysosome (Pankiv et al., 2007). However, many other groups have found that p62 may not be essential for this process (Okatsu et al., 2010), possibly due to redundancy with the related Ub and Atg/LC3II-binding protein NBR1 or other autophagy receptors.

However, this PINK1-Parkin signaling pathway model was challenged by Richard J. Youle’s latest research. They reported that autophagy receptor optineurin (OPTN) and calcium binding and coiled-coil domain 2 (CALCOCO2, also known as NDP52) were the primary receptors for PINK1-and Parkin-mediated mitophagy, while p62 and NBR1 were dispensable for this process (Lazarou et al., 2015). They clarified the respective roles of PINK1 and Parkin in mitophagy, and revealed a new PINK1-dependant/Parkin-independent model of mitophagy. In this new model, PINK1 was not just an initiator for recruiting Parkin, but was a central regulator for inducing mitophagy. PINK1 generated phospho-ubiquitin on mitochondria to recruit autophagy receptor OPTN, NDP52 and upstream autophagy machinery to mitochondria for inducing mitophagy (Lazarou et al., 2015). In other words, Parkin was not essential for this process. In the absence of Parkin, PINK1 induced a lower level of mitophagy owing to the low basal ubiquitin levels on mitochondria; in the presence of Parkin, Parkin served as an amplifier to generate more ubiquitin substrate for PINK1 to phosphorylate, inducing robust and rapid mitophagy induction (Lazarou et al., 2015). Whether other E3 ubiquitin ligases can have the same effects like Parkin needs further exploration.

### NIX and Bnip3 signaling pathway in mitophagy

Bnip3, Bcl-2/E1B-19 KD interacting protein3, is a pro-apoptotic mitochondrial protein. NIX/Bnip3L is a homolog of Bnip3 and they share about 55% amino acid sequence identity (Chen et al., 1999). The C-terminal transmembrane domains of Bnip3 and NIX are inserted into the outer mitochondrial membrane, while their N terminals are exposed to the cytoplasm.

Many groups have found that Bnip3 and NIX play important roles in autophagy and mitophagy. Bnip3 and NIX can increase the production of ROS to activate autophagy (Scherz-Shouval and Elazar, 2011); competition by Bnip3 or NIX for binding Bcl2 could dissociate the Bcl2-Beclin1 complex to release Beclin1 to activate autophagy and mitophagy (Bellot et al., 2009; Maiuri et al., 2007); During *in vivo* starvation, Foxo3 was activated to bind to Bnip3 and NIX promoter regions, which increased their protein level to induce autophagy and mitophagy (Mammucari et al., 2007).

Despite the overall functional similarity between Bnip3 and NIX, many studies have also revealed their differences. Up-regulation NIX could not restore the defects of mitophagy caused by loss of Bnip3 (Shi et al., 2014). Bnip3 induced mitochondrial permeability transition (MPT) and cytochrome c release from isolated mitochondria (Kim et al., 2002), while NIX also induced cytochrome c release but did not change the MPT (Diwan et al., 2007). Bnip3 transcription could be increased by hypoxia but not phenylephrine; in contrast, NIX transcription was increased by phenylephrine but not by hypoxia (Galvez et al., 2006). Under hypoxic conditions, HIF-α bound to the Bnip3 promoter and increased the expression of Bnip3, which promoted mitophagy to remove damaged mitochondria and thereby prevented increase in the levels of ROS (Zhang et al., 2008). Nevertheless, NIX seems to have more important roles in programmed mitochondrial clearance.

NIX plays important roles in red blood cell differentiation and maturation. In most mammals, reticulocytes lack mitochondria, which are achieved by mitophagy regulated by NIX (Aerbajinai et al., 2003; Sandoval et al., 2008; Schweers et al., 2007). In the absence of NIX, the removal of mitochondria was significantly blocked which might be due to the defects in forming mitophagosome (Schweers et al., 2007). Mitochondria uncoupler treatment could restore the defects of mitophagy in NIX deficient erythroid cells, indicating that one mechanism for NIX inducing mitophagy was probably due to its role in regulating mitochondria membrane potential (Sandoval et al., 2008). One very interesting report revealed that BH3-like domain was dispensable for mitochondrial clearance, and mutation of the LIR (LC3-interaction region) had measurable and modest effect on inducing mitophagy, while deletion of the C-terminal of NIX caused complete loss of activity of NIX (Zhang et al., 2012). They identified a minimal essential region (named MER, amino acids 70-86), which was a small domain in its cytoplasmic domain containing hydrophobic amino acid residues and flanking charged residues, for NIX activity (Zhang et al., 2012). However, the exact of role of MER and how MER regulating mitochondrial clearance are still unclear.

### FUNDC1 signaling pathway in mitophagy

FUN14 domain containing 1 (FUNDC1), a mitochondria outer membrane protein, has been reported to play essential role in mitophagy, especially in hypoxic conditions (Liu et al., 2012), but not limited to hypoxic conditions (Chen et al., 2014). FUNDC1 contains a classical LC3-interacting region (LIR) which makes it possible to directly bind with LC3 or ATG8 to induce subsequent mitophagy (Liu et al., 2012). Knockdown of FUNDC1 or mutated the LIR motif could inhibit mitophagy (Liu et al., 2012). Under normoxia conditions, FUNDC1 existed in ‘closed’ form, phosphorylated at Tyr18 by Src tyrosine kinase with a lower affinity binding with LC3 (Liu et al., 2012). Under hypoxia or carbonyl-cyanide p-trifluoromethoxyphenylhydrazone (FCCP) treatment, Src was inactivated and FUNDC1 could be dephosphorylated at serine 13 (Ser-13) by PGAM5 phosphatase through direct interaction to activate FUNDC1 (Chen et al., 2014; Liu et al., 2012). Interestingly, ULK1 could interact with FUNDC1 and phosphorylate it at serine 17, which in contrast to p-Tyr18 enhanced FUNDC1 activity to bind to LC3; once fully activated, FUNDC1 recruited more ULK1 to damaged mitochondria to form a feedback loop to induce mitophagy (Wu et al., 2014). FUNDC1 is also involved in mitochondrial dynamics regulation through interaction with DRP1 and OPA1 (Chen et al., 2016) or calnexin and DRP1 (Wu et al., 2016). FCCP treatment dissociated FUNDC1/OPA1 complex while enhanced DRP1 recruitment to mitochondria to promote mitochondria fission (Chen et al., 2016). Under hypoxic conditions, the association of FUNDC1 and calnexin was attenuated while the interaction between FUNDC1 and DPR1 was enhanced to promote mitochondria fission (Wu et al., 2016).

### The methods to detect mitophagy

Mitophagy is a selective form of autophagy in which damaged mitochondria are degraded. A variety of biochemical and cell biological methods for monitoring mitophagy have been reported. Although existing mitophagy detecting assays have their advantages, one single mitophagy detecting assay cannot accurately identify this process. In practice, complementary methods must be utilized to accurately characterize the process of mitophagy (Zhu et al., 2011).

#### **Fluorescence microscopy**

Observing mitochondria-related proteins or structures with fluorescence microscopy has been widely used in the studies of mitophagy (Dolman et al., 2013). Because mitophagy is a selective form of autophagy, examining the co-localization of mitochondria and GFP-LC3 positive autophagosomal structures is an effective method for understanding it (Hollville et al., 2014). Labeling mitochondria with MitoTracker dyes is a way to observe mitochondria in live cells; however, it is worth noting that different MitoTracker dyes have special characteristics. Some MitoTracker dyes, such as MitoTraker^®^Orange CMTMRos and MitoTraker^®^Red CMXRos, are mitochondria membrane potential-sensitive, and they lose the staining ability after mitochondria depolarization; While others, such as MitoTraker^®^Green and MitoTraker^®^RedFm, accumulate on mitochondria regardless of mitochondrial membrane potential. For fixed cells, labeling mitochondria by staining mitochondria proteins with antibodies, such as TOM20, Cytochrome c and Hsp60, is an effective way to monitor mitochondria-independent mitochondria membrane potential. The percentage of mitochondria and LC3 co-localized puncta provides the quantitative information about mitophagy.

Because damaged mitochondria are finally degraded by lysosomes, the co-localization of mitochondria and lysosomal markers, such as Lysotracker or lysosomal-associated membrane proteins (e.g., LAMP-1 and LAMP-2), could be used to monitor the process of mitophagy.

In Parkin/PINK1-dependent mitophagy pathways, PINK1 is stabilized on depolarized mitochondria and subsequently recruits Parkin from cytoplasm to mitochondria. Parkin then ubiquitylates the mitochondria substrates; while, at the same time, p62, acting as an adaptor, links the ubiquitied mitochondria to autophagosome. Therefore, observing co-localizations of PINK1, Parkin, Ub and p62 with mitochondria through fluorescence microscopy can provide useful information for understanding the process of mitophagy (Hollville et al., 2014; Murakawa et al., 2015; Narendra et al., 2010a).

Disappearance of fluorescence-tagged mitochondrial proteins is a supporting evidence for mitochondrial clearance; however, partial degradation of proteins without mitochondrial removal and mitochondrial clearance through alternative pathways can also result in elimination of fluorescence. Thus tracking multiple proteins in parallel or additional morphological identification must be carried out for confirmation. In addition, reverse confirmation with lysosomal inhibitors is expected to produce increased fluorescence signals.

#### **Immunoblot for mitochondria proteins**

Accompanying the removal of damaged mitochondria by mitophagy, the level of mitochondria proteins, such as TOM20 (outer mitochondrial protein), TIM23 (inner mitochondrial protein), cytochrome C oxidase subunit II (COXII) (inner mitochondrial protein) and Cytochrome c (intermembrane space protein) will decrease. Western blot analysis for these proteins is another quantified method to assess the process of mitophagy. However, this method should be combined detection of different mitochondrial proteins, including outer and inner mitochondria proteins, intermembrane space protein, and matrix protein (Ding and Yin, 2012; Lazarou et al., 2015). Only detection of mitochondrial outer proteins, such as TOM20 and MFN1/2, is misleading because these proteins are also degraded via the proteasome system (Lazarou et al., 2015).

**Figure 3:**
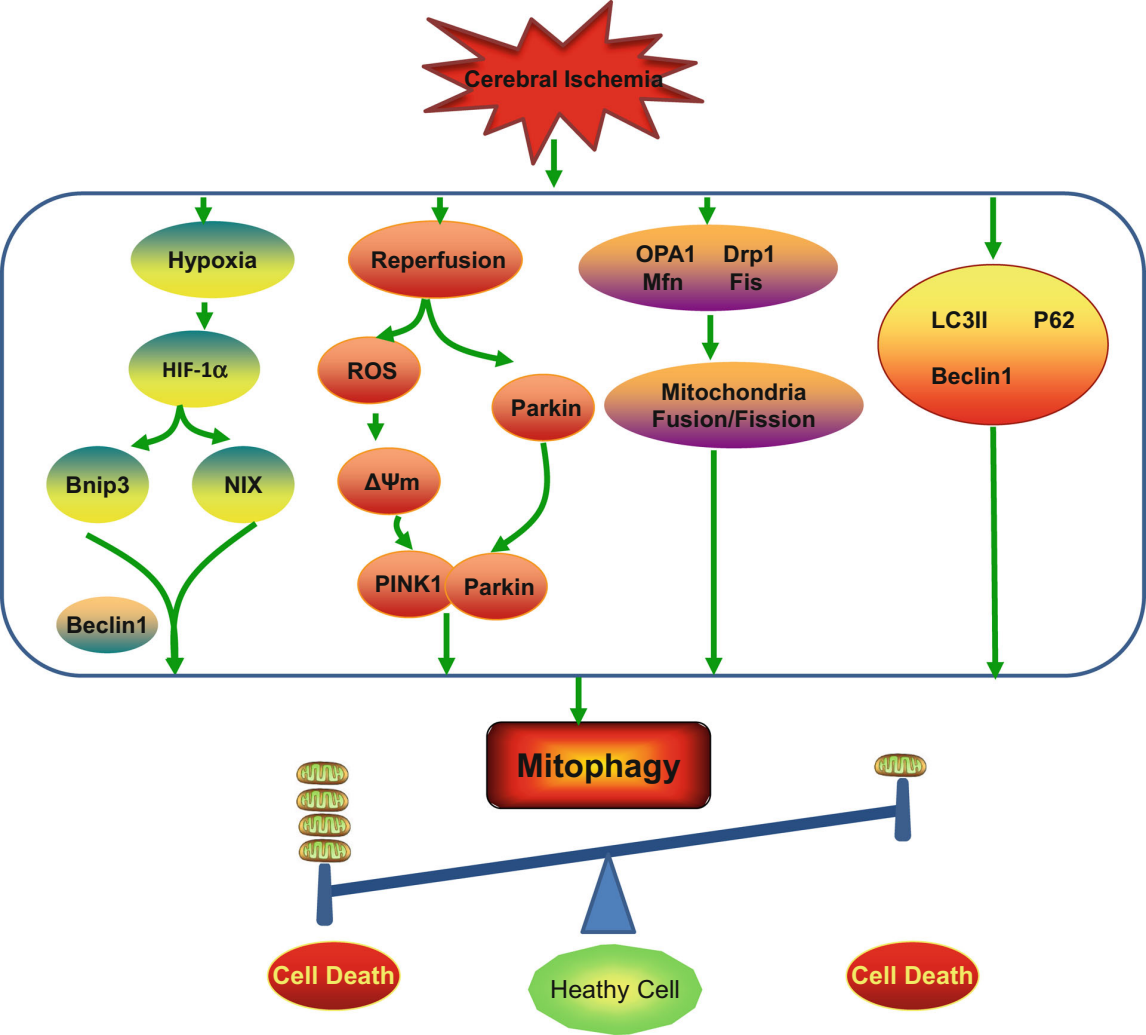
**Possible mitophagy signalling pathways involved in cerebral ischemia**. During the process of cerebral ischemia, many different signalling pathways are involved in the activation or suppression of mitophagy. (1) The process of cerebral ischemia can cause hypoxic conditions in tissue, which can increase the Bnip3 and NIX levels, and cause release of Beclin1 from the Bcl-2-Beclin1 complex, and finally induce mitophagy. (2) Reperfusion is the most effective therapy for cerebral ischemia. Reperfusion increases the level of ROS, which can decrease the mitochondria membrane potential, and lead to the translocation of Parkin from cytosol to damaged mitochondria. Then, mitophagy can be facilitated in Parkin-dependent or Parkin-independent ways. (3) Mitochondrial fragmentation and fission are essential for mitophagy. Cerebral ischemia can decrease the level of mitochondrial fusion proteins, such as Opa1 and Mfn2, and increase the level of mitochondrial fission proteins, such as DRP1 and Fis. (4) Rapamycin can significantly increase the expression of LC3-II and Beclin1, and promote the translocation of P62 to damaged mitochondria, and finally facilitate mitophagy to exhibit neuroprotective functions. The process of mitophagy must be restricted to dysfunctional mitochondria and kept at a balanced level. Insufficient removal damaged mitochondria or excessive degradation of essential mitochondria will both cause cell death

#### **Mitochondrial DNA quantification**

Mitochondria, as isolated organelles, have a small amount of their own DNA. Mitochondria DNA (mtDNA) contains 37 genes which are essential for mitochondrial function (Taanman, 1999). The number of mtDNA is reduced companied by mitophagy, while the nuclear DNA (nDNA) are relatively stable. The ratio change between mtDNA and nDNA can be an indicator of mitophagy (Lazarou et al., 2015; Murakawa et al., 2015). The Human Mitochondrial DNA (mtDNA) Monitoring Primer Set (Takara) is used for amplification of mtDNA by real-time PCR and using nDNA content as a standard (Murakawa et al., 2015); relative quantification of mtDNA is analyzed by the difference in Ct values for mtDNA and nDNA. Another mitochondrial DNA quantification method is using DNA immunostaining and quantified by immunofluorescence. The total cellular DNA volume (cDNA_v_) (cDNA fluorescence intensity) is determined by anti-DNA antibodies and nuclear DNA stain volume (nDNA_v_) (nDNA fluorescence intensity) is determined by using DAPI. The percentage of mtDNA stain remaining indicates mitophagy level, which could be calculated using this formula: (cDNA_v_ − nDNA_v_)/N (N is the cell numbers) (Lazarou et al., 2015).

#### **Ultrastructural evaluation**

Electron microscopy (EM) provides direct images of autophagosomes with engulfed mitochondria. Analysis of autophagosome inclusions by comparing their morphology with other cytosolic organelles helps to identify mitochondria existing in these structures (Zhu et al., 2011). EM could provide the ‘seeing is believing’ data for assessing the process of mitophagy (Ding and Yin, 2012; Murakawa et al., 2015). However, because of the limited cell numbers or sections, data from EM studies must be interpreted carefully and rigorously.

#### **Detection of mitophagy using mitochondrial-targeted mKeima**

Recently, a sensitive and quantitative technique has been developed to visualize the process of mitophagy. This technique uses the mitochondrial-targeted mKeima to detect and analyze mitophagy (Bingol et al., 2014; Kageyama et al., 2014; Katayama et al., 2011; Murakawa et al., 2015; Nezich et al., 2015). Keima is a coral-derived acid-stable lysosomal protease-resistant fluorescent protein that exhibits a reversible change in color in response to acidic pH. Keima exhibits a bimodal excitation spectrum peaking at 440 and 586 nm, respectively corresponding to the neutral and ionized states of chromophore’s phenolic hydroxyl moiety (Violot et al., 2009). These paradoxical pH-dependent changes in fluorescence make Keima able to clearly track the conversion of autophagosome to autolysosome (Katayama et al., 2011). To visualize the process of mitophagy, mitochondrial targeted monomeric Keima (mt-mKeima) was generated by fusing a tandem repeat of the COX VIII pre-sequence to mKeima to localize the protein mKeima to the matrix of mitochondria (Katayama et al., 2011). After treatment with CCCP, cells exhibited strong signals for mt-mKeima at an excitation wavelength of 550 nm, while the control groups displayed strong fluorescence at 438 nm (Katayama et al., 2011). The ratio of (550/438) can be used to clearly demonstrate the different distribution of intact (green) and degraded (red) mitochondria (Katayama et al., 2011). After cells were treated with CCCP for 2 h, punctate structures with green fluorescence emerged, indicating that EGFP-Parkin had been recruited to impaired mitochondria. This emergence was accompanied by the appearance of a steadily increasing ratio (550/438) of signals from mt-mKeima (Katayama et al., 2011). The mt-mKeima method is also used to measuring mitophagy *in vivo* (Sun et al., 2015). Mitophagy basal levels are different in different tissues, with a low signal in thymus and high rates in heart and brain (Sun et al., 2015). In one study, the Purkinje cell layer and the cerebellum regions had a higher level of mitophagy, while the cortex, striatum and substantia nigra regions had only modest levels of basal mitophagy (Sun et al., 2015).

## MITOPHAGY AND CEREBRAL ISCHEMIA

As mentioned above, mutations of PINK1 and Parkin are suspected to be pathological causes of some forms of Parkinson’s disease in that they result in defects in mitophagy. In addition, some other neurodegenerative diseases, including Alzheimer’s disease (AD) (Casley et al., 2002) and Huntington’s disease (HD) (Cui et al., 2006), are also related to mitophagy. Although currently there is no systematic theory to explain the role of mitophagy in cerebral ischemia, more and more groups believe mitophagy may have an important role in the process of cerebral ischemia. The effects of mitophagy on cerebral ischemia brain injury have been controversial for years (Table 1) (Li et al., 2012, Zuo et al., 2014). Both insufficient removal of damaged mitochondria or excessive degradation of essential mitochondria will cause cell death (Ordureau and Harper, 2014; Shi et al., 2014), and the possible mitophagy pathways involved in cerebral ischemia are summarized in Fig. 3 and discussed in the following sections.

**Table 1:** **Highlights of research findings on the roles of mitophagy in cerebral ischemia**

Animal/Cell	Model	Results	Effect of mitophagy	Reference
Male C57BL/6 mice Primary cortical neurons	tMCAO OGD-Rep	Cerebral ischemia-reperfusion induces mitophagy by causing Parkin translocation from cytosol to mitochondria.	Protective	Zhang et al. (2013)
Male Sprague-Dawley rats NeuN positive cells	tMCAO	Rapamycin induces mitophagy and attenuates mitochondrial dysfunction.	Protective	Li et al. (2014)
Male Sprague-Dawley rats (Brain tissue) PC12 cells	pMCAO OGD	Inhibition of DRP1 by pharmacologic inhibitor or siRNA increases the infarct volume and aggravates neurological deficits.	Protective	Zuo et al. (2014)
Male C57BL/6 mic**e** Primary cortical neurons	tMCAO OGD-Rep	Endoplasmic reticulum (ER) stress induced by ER stress activators (tunicamycin and thapsigargin) protect against transient ischemic brain injury through Parkin-dependent mitophagy suppression.	Protective	Zhang et al. (2014)
Male Sprague-Dawley rats CA3 neurons Primary hippocampal neurons	IPC OGD	Suppression of DRP1 increases the vulnerability of cells to OGD and global ischemia due to amplified mitochondria mediated injury.	Protective	Zuo et al. (2016)
Male C57BL/6J mice Cortex tissue Cortex neuron	MCAO	Hyperglycemia enhances ischemia-induced mitochondrial dynamic imbalance towards fission.	Harmful	Kumari et al. (2012)
Male C57BL/6J mice NeuN positive cells Murine hippocampal neuronal HT22 cells	MCAO	Selenium prevents glutamate and hypoxia-induced cell death by reducing glutamate-induced ROS production and preserving mitochondrial membrane potential and increasing mitochondrial biogenesis.	Harmful	Mehta et al. (2012)
Male Wistar rats Coronal cortex tissue	MCAO	Reperfusion promotes mitochondrial dysfunction by decreasing mitochondria membrane potential.	Harmful	Li et al. (2012)
Male Sprague-Dawley rats Primary cortical neurons	MCAO	Carnosine attenuates the increase of p-DRP1 and Parkin to inhibit the process of mitophagy.	Harmful	Baek et al. (2014)
Male Wistar rats Hippocampal neurons	MCAO	Resveratrol significantly increases SOD activity and prevents the loss of mitochondria membrane potential.	Harmful	Wang et al. (2014)
Mice Primary cortical neurons	Neonatal stroke mode OGD-Rep	BNIP3 triggers excessive mitophagy.	Harmful	Shi et al. (2014)

### Mitochondrial damage in cerebral ischemia

Energy consumption by the brain is huge compared to its relative volume, while energy storage capacity is low. Therefore lack of blood supply accompanied by oxygen and glucose insufficiency for even a short time period may cause severe damage to the brain. There are profound reductions in ATP levels for very short periods during ischemia. Mitochondria play a key role in various cell processes including ATP production, Ca^2^^+^ homeostasis, reactive oxygen species (ROS) generation, and apoptosis. Thus, functional alterations in mitochondria therefore have enormous potential for causing severe cell damage and play important roles in the pathophysiological process of cerebral ischemia.

The reductions of ATP levels and accumulation of AMP activate the adenosine monophosphate AMP-activated protein kinase (AMPK) (Yan et al., 2013). AMPK, a serine threonine kinase, serves as a central metabolic sensor. AMPK can directly phosphorylate multiple downstream substrates to inhibit ATP-consuming biosynthetic pathways and promote catabolic ATP regenerating processes to restore intracellular energy levels. During ischemic injury, activated AMPK suppressed mammalian target of rapamycin (mTOR) by phosphorylation, thereby up-regulate autophagy, to play a protective effects on cell survival during ischemia (Takagi et al., 2007). AMPK phosphorylated ULK1 and activated ULK1 to induce mitophagy, to maintain mitochondria homeostasis and cell survival during starvation (Egan et al., 2011). In addition, AMPK could directly regulate mitochondria biology through phosphorylation of mitochondrial fission factor (MFF) (Toyama et al., 2016). MFF was a dominant receptor for DRP1 (Loson et al., 2013; Otera et al., 2010; Shen et al., 2014). AMPK activated MFF to recruit more DRP1 to mitochondria to induce mitochondria fission and mitophagy (Toyama et al., 2016). Another report revealed that ATP could directly regulate PINK1-Parkin dependent mitophagy (Lee et al., 2015). All of these make AMPK as a potential therapeutic target for brain stroke.

Moreover, mitochondria are the primary intracellular sources of ROS and evidence obtained over the past two decades reveals that reactive oxygen species (ROS) are related to brain lesions. In normal conditions, ROS generation and elimination are balanced. Small amounts of ROS are generated in the form of ${\text{O}}_{2}^{ \bullet - }$ and then converted into H_2_O_2_ by the antioxidant enzyme, magnesium superoxide dismutase (Chan, 2001). Under the conditions of cerebral ischemia, ROS is overproduced by mitochondria, which directly damages lipids, proteins and nucleic acids, leading to cell injury and cell death (Chan, 2001). ROS is also involved in reperfusion injury (Yoshida et al., 1982). Resveratrol has been reported to be a unique antioxidant compound and extensive research has revealed resveratrol protects against stroke in animal models (Sinha et al., 2002). Resveratrol could significantly increase SOD activity and improve mitochondrial integrity by preventing the loss of mitochondria membrane potential to reduce the neuron apoptosis after cerebral ischemia (Wang et al., 2014). Selenium prevented glutamate and hypoxia-induced cell death through reducing glutamate-induced ROS production, preserving mitochondrial membrane potential, and increasing mitochondrial biogenesis (Mehta et al., 2012). Consistently, pre-treatment with antioxidant (Mitochondria-targeted antioxidant 10-(6-plastoquinonyl) decyltriphenyl-phosphonium) prevented ischemia induced injury (Plotnikov et al., 2008). Methylene blue, another mitochondrial targeted antioxidant also played neuroprotective role in cerebral ischemia, through regulating mitophagy (Di et al., 2015) (Mechanisms will be discussed in the later part).

Interestingly, moderately elevated ROS levels can induce mitophagy in a DRP1 dependent manner (Frank et al., 2012). In P53 or TP53-induced glycolysis (TIGAR) knockout mice, ROS levels was increased via reduction of NADPH and glutathione (GSH) and followed by Bnip3 activation to induce mitophagy against ischemic injury; antioxidant N-acetylcysteine could block this mitophagy process which indicated ROS was required in this adaptive response (Hoshino et al., 2012).

### Classic mitophagy signaling pathways involved in cerebral ischemia

Reperfusion can promotes mitochondrial dysfunction following focal cerebral ischemia in rats. During the reperfusion period, the membrane potential of mitochondrial is decreased significantly; this decrease is the main cause of mitophagy (Li et al., 2012). It has also been established that mitophagy can be induced in the cerebral ischemia reperfusion (I-R) process both in *in vivo* and *in vitro* models, and that this process can be reversed by 3-methyladenine and Atg7 silencing (Zhang et al., 2013). Studies have also demonstrated that Parkin could be translocated to mitochondria during reperfusion and that ischemia-induced neuronal injury was aggravated after administration of mitophagy inhibitor mdivi-1 in the reperfusion phase, suggesting that mitophagy underlies the neuroprotection that occurred in the process of cerebral ischemia reperfusion (Zhang et al., 2013). Zhang et al. also reported that endoplasmic reticulum (ER) stress induced by ER stress activators (tunicamycin and thapsigargin) protected against the transient ischemic brain injury through Parkin-dependent mitophagy (Zhang et al., 2014). The neuroprotective effects of ER stress activators have been shown to be reversed by autophagy inhibition (3-methyladenine and Atg7 knockdown) or Parkin silencing (Zhang et al., 2014). In one transient focal cerebral ischemia model, Parkin protein level was decreased further companied by increasing reperfusion time after 1 h left middle cerebral artery (MCA), while neuronal cell injury was increased companied by Parkin’s reduction (Mengesdorf et al., 2002), which might be due to defective mitophagy.

Despite of PINK1-Parkin mitophagy pathway, under hypoxic conditions, Bnip3 and NIX were activated at the mRNA level in a HIF-α dependent manner (Bruick, 2000; Zhang et al., 2008). It has also been reported Bnip3 was involved in delayed neuronal death in stroke (Shi et al., 2014). When primary cortical neurons were treated with OGD for 6 h followed by different periods of reperfusion (24, 48, 72 h), the Bnip3 protein level increased accompanied by increased delayed neuronal loss, which appeared to be due to Bnip's triggering of excessive mitophagy (Shi et al., 2014). Although Bnip3 protein level was increased both *in vitro* (Bruick, 2000) and *in vivo* (Schmidt-Kastner et al., 2004), NIX protein seemed no significant increase. In contrast, Brise-Archbold, J.L. et al. reported that NIX protein level was increased after 4 to 7 days hypoxia/serum deprivation in cultured Chinese hamster ovary cells (CHO-K1) and NIX translocated to mitochondria after 5 days hypoxia/serum; while upregulation and translocation of NIX were observed after 6 h of middle cerebral artery occlusion in the rat model (Birse-Archbold et al., 2005). *In vivo*, NIX was activated (upregulation and translocation) before histological damage (infarct development, neuronal loss) or biochemical marker (Bax activation or caspase-3 activation) were detected, which indicated NIX might be a potential therapeutic target in ischemic injury (Birse-Archbold et al., 2005). Interestingly, different from many studies, NIX as mitochondria out membrane protein (Chen et al., 1999; Ding et al., 2010; Kanki, 2010), they reported NIX was predominately a cytosolic protein which translocated from cytosol to mitochondria after hypoxia stress (Birse-Archbold et al., 2005); in addition, the mitophagy role in this study could be further explored.

### Mitochondria dynamics, mitophagy, cerebral ischemia

Mitochondrial dynamics, the processes including mitochondrial fusion, fission, biogenesis and mitophagy, have been recently implicated in cerebral ischemia injury. Mitochondria are isolated organelles constantly going through cycles of fusion and fission. Mitochondrial fusion is mediated by dynamin-related GTPases termed mitofusins (Mfn1 and Mfn2) and optic atrophy protein 1 (Opa1). Conversely, mitochondrial fission is regulated by mitochondrial fission 1 protein (Fis1) and the dynamin-related protein 1 (DRP1) (Chen and Chan, 2010; James et al., 2003; Yamamori et al., 2015; Zuo et al., 2014). Fission can generate two daughter mitochondria with either increased or decreased membrane potential; the depolarized daughter mitochondria cannot be fused and have to be removed by mitophagy, while overexpression of Opal leads to increased mitochondria fusion and decreased mitophagy (Twig et al., 2008). Fragmented mitochondria are more likely to be taken up by autophagosome due to their smaller size and can facilitate mitophagy (Gomes et al., 2011). Santosh Kumari et al. revealed that cerebral ischemia could decrease the level of mitochondrial fusion proteins Opa1 and Mfn2, which were essential for mitochondrial fusion (Kumari et al., 2012). They also demonstrated that pre-ischemic hyperglycemia could increase the level of fission proteins DRP1 and Fis1 (Kumari et al., 2012). Hyperglycemia tended to tip the ischemia-induced mitochondrial dynamic balance towards fission, which led to mitochondrial fragmentation and damage (Kumari et al., 2012). It has been reported that carnosine, an endogenous pleiotropic dipeptide which has neuroprotective activity against ischemic brain damage, could attenuate the increase of p-DRP1 and Parkin to inhibit the process of mitophagy (Baek et al., 2014). However, another report has revealed that inhibition of DRP1 by pharmacologic inhibitor or siRNA resulted in increasing the infarct volume and aggravating the neurological deficits in a rat model of pMCAO (permanent middle cerebral artery occlusion). These effects may be due to the change of ROS generation, Cyt-c release and activation of caspase-3 (Zuo et al., 2014). In hippocampal CA3 neurons, ischemia induced more mitophagy; this was accompanied by increasing DRP1 levels. Suppression of DRP1 increased the vulnerability of cells to OGD and global ischemia due to amplified mitochondria-mediated injury (Zuo et al., 2016). All of these indicate that DRP1 can be a potential therapeutic target for brain ischemic stroke.

### Compounds that potentially regulate mitophagy in cerebral ischemia

Rapamycin is known to exhibit neuroprotective functions via the activation of autophagy, as shown in a cerebral ischemia model (Malagelada et al., 2010). Li et al. reported that rapamycin could reduce brain injury after cerebral ischemia by promoting mitophagy (Li et al., 2014). Their results demonstrated that rapamycin could significantly increase the expression of L3-II and Beclin-1 level, which means increased autophagy and mitophagy (Li et al., 2014). This process is thought to be mediated by upregulating p62 translocation to the mitochondria in response to ischemia injury, which led to reduced infarct volume and inhibition of mitochondrial dysfunction (Li et al., 2014).

Methylene blue (MB) is a lipophilic compound and has been demonstrated to play neuroprotective roles in cerebral ischemia-reperfusion injury (Miclescu et al., 2010; Shen et al., 2013; Wen et al., 2011). In a MCAO model, MB improved neurological function and reduced the infarct volume after acute cerebral ischemia due to augmenting mitophagy (Di et al., 2015). In a *vitro* OGD model, they revealed MB promoted mitophagy by maintaining the MMP at a relatively high level (Di et al., 2015). However, mitophagy is usually induced by the loss of MMP by regulating Parkin/PINK1 mitophagy pathway or mitochondria fission and fusion (Jin et al., 2010b; Kondapalli et al., 2012; Matsuda et al., 2010; Narendra et al., 2010b; Nguyen et al., 2016; Youle and Narendra, 2011).

### Mitophagy, inflammation response, cerebral ischemia

The inflammation response is an important mechanism in the pathogenesis of cerebral ischemia and other forms of ischemic brain injury. Ischemic injury will be amplified with an acute and prolonged inflammatory response, characterized by activation of inflammatory cells (McColl et al., 2007). Reintroduction of blood into ischemic tissue will also cause a strong release of inflammatory mediators like tumor necrosis factor (TNF) and leukocyte-endothelial cell adhesion molecules; all these cellular events can initiate an inflammatory condition which may contribute to further vascular dysfunction and stroke damage (Carden and Granger, 2000; Jin et al., 2010a; Prestigiacomo et al., 1999; Ritter et al., 2000). Therefore, anti-inflammatory strategies have been proposed (Gao et al., 2013; Palencia et al., 2015; Zhang et al., 1995). Interestingly, recently, several studies have identified new roles for mitochondria and mitophagy in the regulation of an-inflammatory processes (Matheoud et al., 2016; Mills and O’Neill, 2016; Minton, 2016; Zhong et al., 2016). Mitochondria are central regulators of pyrin domains-containing 3 (NLRP3) inflammasome’s activation (Gurung et al., 2015); inflammasome is a molecular platform to activate innate immune defense and pyroptosis through several pro-inflammatory cytokines and its interaction with caspase-1 (Schroder and Tschopp, 2010; Strowig et al., 2012). Inflammasome can be activated by damaged mitochondria through regulating reactive oxygen species (ROS), Ca2+ overload, reduced NAD^+^, mtDNA and so on (Gurung et al., 2015). Anti-inflammatory response by regulating mitophagy has attracted more attention. Zhong et al. reported that Nuclear factor-κB (NF-κB), a key activator of inflammation by priming NLRP3 activation, could restrain NLRP3 activation by regulating p62-dependent mitophagy, thus to prevent excessive tissue damage to the host. Zhao et al. revealed that A151, a synthetic oligodeoxynucleotide containing multiple telomeric TTAGGG motifs could reduce ischemic brain damage and NLRP3 protein level, and A151 could maintain mitochondrial membrane potential intact, which indicated a role of mitochondria in A151’s suppression of inflammation and protection of ischemic injury (Zhao et al., 2015). Collectively, accumulating knowledge about mitophagy or mitochondria’s participation in the processes of an-inflammatory responses, regulating mitophagy in cerebral ischemia will become a potential therapeutic strategy through anti-inflammatory process.

## **CONCLUSIONS**

Mitochondria are the essential organelles which provide energy to cells by producing ATP. Removal of damaged or dysfunctional mitochondria by mitophagy has been proved to be an important mitochondrial quality control mechanism. Although mitophagy has no unified role in cerebral ischemia, great progress has been achieved in the research on the functions of mitophagy in cerebral ischemia. Much evidence from recent research supports the belief that mitophagy has a neuroprotective role in cerebral ischemia, at least to a certain extent. However, many questions remain unanswered. For example, how can we exactly monitor mitophagy during the cerebral ischemia process? How does Parkin translocate from cytosol to mitochondria after cerebral ischemia? What are the roles of PINK1 and FUNDC1 in the process of cerebral ischemia? In addition, it is also important to study the roles of mitophagy in different phases or types of cerebral ischemia. Answering these questions will not only improve our understanding of the relationship of stroke and mitophagy, but it will also provide theoretical support to help people find new effective treatments for stroke patients.

## **ACKNOWLEDGEMENTS**

This work was funded by the Faculty Research Grant of Hong Kong Baptist University (FRG2/15-16/022) and the Guandong Natural Science Foundation (2014A030313766 and 2016A030313008).

## **COMPLIANCE WITH ETHICS GUIDELINES**

Yan-Cheng Tang, Hong-Xia Tian, Tao Yi and Hu-Biao Chen declare they have no conflict of interest. This article does not contain any studies with human or animal subjects performed by the any of the authors.
